# Acute Portomesenteric Venous Thrombosis following Laparoscopic Small Bowel Resection and Ventral Hernia Repair

**DOI:** 10.1155/2015/851852

**Published:** 2015-08-03

**Authors:** Bhradeev Sivasambu, Meera Yogarajah, Thomas Wilson

**Affiliations:** Department of Medicine, Interfaith Medical Center, Brooklyn, NY 11213, USA

## Abstract

Acute portomesenteric venous thrombosis is a rare but life-threatening complication of laparoscopic surgery that has been described in literature. Prompt diagnosis and early initiation of treatment are vital to prevent life-threatening complications such as mesenteric ischemia and infarction. A 51-year-old lady had laparoscopic small bowel resection and primary anastomosis with ventral hernia repair 4 weeks earlier for partial small bowel obstruction. Her postoperative period was uneventful and she was discharged home. Four weeks after surgery she developed watery diarrhea and generalized abdominal pain for four-day duration. A computed tomography of the abdomen revealed portomesenteric venous thrombosis although a computed tomography of abdomen before surgery 4 weeks back did not show any portomesenteric venous thrombosis. We are reporting a case of acute portomesenteric venous thrombosis as a complication of laparoscopic surgery.

## 1. Introduction

Acute portomesenteric venous thrombosis is an uncommon complication of laparoscopic surgery that has been described more often after laparoscopic bariatric surgeries. The pathogenesis of portomesenteric venous thrombosis associated with laparoscopic procedures is multifactorial. The minimally invasive nature of laparoscopic surgeries has gained increased popularity among both patients and physicians in the recent decades. Hence knowledge on rare postoperative complications becomes vital for practicing physicians to avoid delay in appropriate treatment and life-threatening complications.

## 2. Case Presentation

A 51-year-old lady with recent history of laparoscopic abdominal surgery presented to our emergency department with watery diarrhea and generalized abdominal pain for four-day duration without vomiting, fever, or any other systemic complaints. She denied recent travel and was unable to relate any food that could have triggered her symptomatology. Four weeks earlier she was found to have small bowel obstruction secondary to incarcerated incisional hernia with trapped bowel loop in the hernial sac. She has a history of caesarean section with midline incision. Patient had laparoscopic small bowel resection and primary anastomosis with ventral incisional hernia repair. During her surgery there was no mechanical injury to the portal and mesenteric veins and standard pressure of pneumoperitoneum was utilized. She was perioperatively anticoagulated with prophylactic dose of heparin. Her postoperative period was uneventful with no abdominal sepsis and she was discharged home. Histology of the small bowel revealed submucosal congestion and serosal granulation. Her other medical problems were hypertension, diabetes mellitus, peripheral vascular disease, and meningioma, abnormal mammogram with BI-RADS score of 5 (Breast Imaging-Reporting and Data System) awaiting biopsy. There was no personal or family history of clotting disorders. Physical examination was positive for generalized abdominal tenderness without guarding or rigidity. All other system examination was unremarkable. Initial labs revealed mild neutrophil leukocytosis with a white blood cell count of 12 200/*μ*L and 75% of neutrophils. She also had normochromic normocytic anemia with hemoglobin of 10.2 g/dL and normal platelet count. Chemistry showed normal electrolytes and renal function. Aspartate aminotransferase (AST) was mildly elevated to 84 IU/L (normal range 15–41) and other liver enzymes were normal. Bilirubin, total protein, albumin, and coagulation profile was normal.

**Figure 1:**
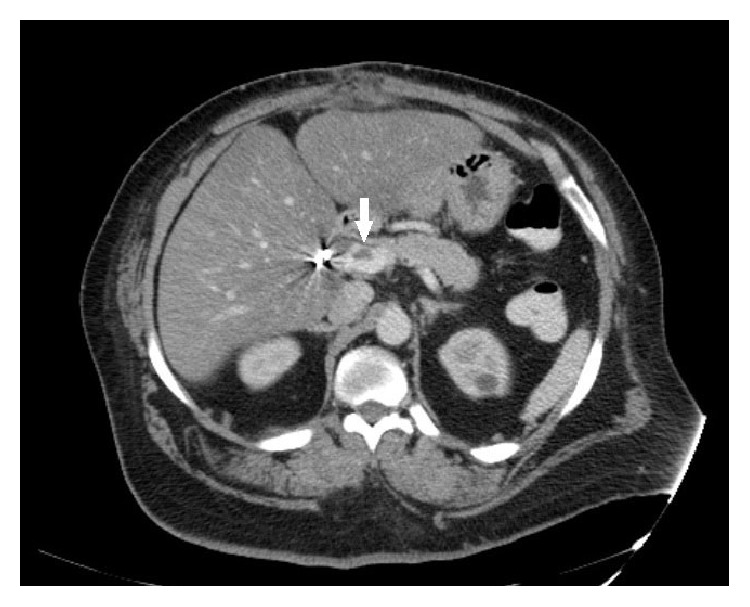
Computed tomography of abdomen and pelvis axial view showing portal vein thrombosis.

**Figure 2:**
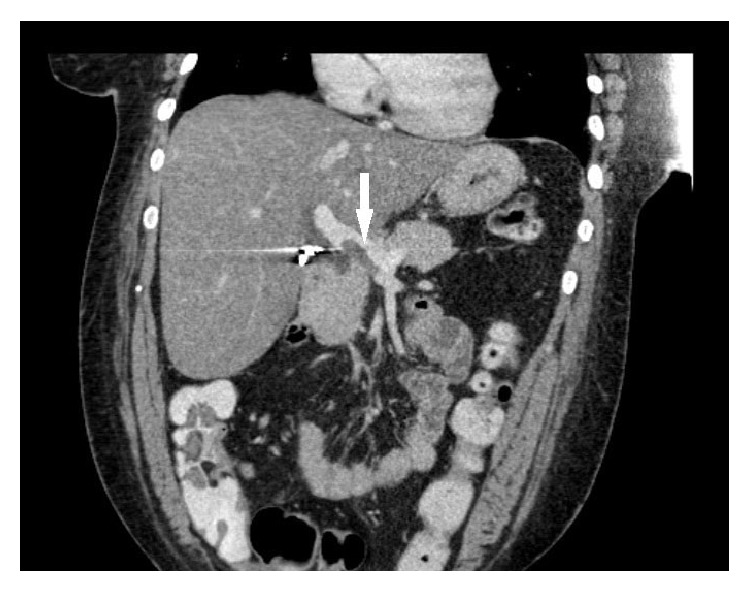
Computed tomography of abdomen and pelvis coronal view showing portal vein thrombosis.

A computed tomography of the abdomen was done to rule out anastomotic leak or intra-abdominal abscess as a postoperative complication and incidentally revealed filling defect within the portal vein and a branch of the superior mesenteric vein compatible with thrombosis (Figures 1 and 2) explaining her clinical symptoms. Computed tomography of her abdomen done before the surgery 4 weeks back did not show any portal or mesenteric vein thrombosis.

During her hospital stay biopsy of the left breast for abnormal mammogram revealed invasive ductal carcinoma in situ which may have predisposed to systemic hypercoagulable state. Another workup for coagulopathy was unremarkable. She had normal protein C and protein S levels with no resistance to activated protein C. Her lupus anticoagulant, anti-cardiolipin antibody, and beta 2 glycoprotein antibody were negative. There was no prothrombin gene mutation and antithrombin 3 levels were normal. Flow cytometry for paroxysmal nocturnal hemoglobinuria was negative. Patient was started on enoxaparin and warfarin and her diarrhea and abdominal pain resolved.

## 3. Discussion

Portomesenteric venous thrombosis is being increasingly recognized due to the frequent use of ultrasonography. The commonest predisposing factor is primary hepatic parenchymal disease [1]. In a healthy liver the etiology could be due to local or systemic causes. Local causes are abdominal inflammatory conditions such as pancreatitis, inflammatory bowel disease, infection, and abdominal surgeries especially with handling of the portal and mesenteric veins such as splenectomy [2]. Systemic causes are prothrombotic states secondary to genetic coagulopathies like protein C and S deficiency, prothrombin gene mutation [3], antithrombin III deficiency, factor V Leiden mutation, and hyperhomocysteinemia or acquired causes like contraceptive use [4], recent pregnancy, antiphospholipid syndrome, myeloproliferative disorders, and paroxysmal nocturnal hemoglobinuria.

Acute portomesenteric venous thrombosis is a rare but serious life-threatening complication of laparoscopic surgery that has been described in literature. A systematic review by James et al. revealed 18 cases of portomesenteric venous thrombosis after laparoscopic surgery excluding splenectomy [5]. Out of these, 7 cases were after Roux-en-Y gastric bypass, 5 cases were after Nissen fundoplication, 3 cases were after partial colectomy [6, 7], 2 cases were after cholecystectomy, and 1 case was after appendectomy.

There have been no reported cases of portomesenteric thrombosis after laparoscopic small bowel resection and ventral incisional hernia repair. The etiology of portomesenteric thrombosis is usually associated with more than 1 risk factor. Our patient was diagnosed with invasive ductal carcinoma in situ of the breast during the hospital stay which could have predisposed for a systemic hypercoagulable state. Breast cancer is commonly associated with deep vein thrombosis and pulmonary embolism; however portal vein thrombosis has not been reported. Our patient is a unique case of portomesenteric thrombosis after laparoscopic small bowel resection and ventral incisional hernia repair with predisposing breast cancer.

The pathogenesis of portomesenteric venous thrombosis in laparoscopic surgery is multifactorial and could be explained by Virchow's triad which includes vascular injury, hypercoagulability, and stagnation of blood flow. The increased intra-abdominal pressure as a consequence of carbon dioxide pneumoperitoneum during the laparoscopic surgery contributes to decrease in portal venous flow [8, 9]. The pressure limit at which this change occurs is unclear; however a pressure of >10 mm/hg showed significant reduction in diameter of the portal venous trunk and the mean portal blood flow and causes stagnation of blood. Moreover the raised intra-abdominal pressure also contributes to the coagulation abnormalities and potentiates hypercoagulability. Other reported contributing factors are patient positioning during the surgery which might impede the portal blood flow and create stasis and direct vascular injury to the portal venous system.

Acute portomesenteric venous thrombosis can be asymptomatic or present nonspecifically with abdominal pain, dyspeptic symptoms, and diarrhea when the superior mesenteric vein is involved. The clinical syndrome may resemble gastroenteritis and the diagnosis can be missed, leading to life-threatening complications such as mesenteric ischemia and infarction. Early diagnosis and prompt initiation of anticoagulation therapy can prevent the sequela of chronic portomesenteric thrombosis and portal hypertension.

The definitive diagnosis is made by imaging with the use of contrast enhanced computed tomography or color Doppler ultrasonography. Other imaging modalities that could be used are angiography or magnetic resonance imaging. The choice of treatment is decided based on the extent of thrombosis and the presence or absence of bowel ischemia. Mostly therapeutic anticoagulation is the treatment of choice. The goal is to prevent elongation of the thrombus and to facilitate recanalization and avoid the sequelae of intestinal infarction and portal hypertension. When a predisposing condition is identified treatment of it is recommended. The duration of treatment remains unclear. Treatment may be lifelong for patients with prothrombotic states and may continue for 6 months in other patients. Low molecular weight heparin or heparin infusion should be started immediately followed by warfarin with a target INR of 2-3. In patients with bowel infarction surgical exploration is necessary and delays can lead to increased mortality. Other modalities described in literature are endovascular thrombolysis and percutaneous thrombectomy which could be utilized in extensive thrombosis [5].

We report the first case of portomesenteric venous thrombosis as a complication of laparoscopic small bowel resection and primary anastomosis with ventral incisional hernia repair with predisposing breast carcinoma induced thrombophilic state and knowledge on this rare complication becomes significant with the increasing number of laparoscopic procedures.
